# Dietary glutamine supplementation improves growth performance, intestinal morphology, and immunity function associated with cecal microbiota alterations in broilers

**DOI:** 10.1016/j.psj.2025.105982

**Published:** 2025-10-18

**Authors:** Xuelan Liu, Shuhang Yin, Chunyan Fu, Xia Li, Peipei Yan, Heng Zhang, Yan Shang, Tianhong Shi, Qingtao Gao

**Affiliations:** a Poultry Institute, Shandong Academy of Agricultural Sciences, Jinan 250100, China; b Shandong Provincial Key Laboratory of Livestock and Poultry Breeding, Shandong Academy of Agricultural Sciences, Jinan 250100, China

**Keywords:** Glutamine, Growth performance, Intestinal health, Broilers

## Abstract

Broilers face challenges in improving intestinal health and growth performance. Glutamine (Gln), a functional amino acid, exerts beneficial effects in promoting intestinal development and immunity. This study investigated the effects of graded levels of dietary Gln supplementation on the growth performance, intestinal morphology, immune indices, and cecal microbiota of broilers. Three hundred one-day-old Arbor Acres broilers (51.67±1.42 g) were assigned to five groups in a completely random design, with six replicates each group and 10 broilers in each replicate. The control group was fed the basal diet (Con), while the experimental groups were fed the basal diet containing 4 g/kg (Gln1), 6 g/kg (Gln2), 8 g/kg (Gln3), and 10 g/kg Gln (Gln4), respectively. The results showed that dietary Gln supplementation had quadratic effect on ADG, F/G, and final BW of broilers (*P* < 0.01), with higher average daily gain (ADG), final body weight (BW) in Gln2 group (*P* < 0.05). Dietary Gln supplementation quadratically improved serum immune indices of broilers (*P* < 0.01), with higher IgA, IgG, and IgM levels in Gln1 and Gln2 groups (*P* < 0.05). The Gln1 and Gln2 groups increased Bursa of Fabricius index at both d21 and d42 (*P* < 0.05). There was a quadratic effect of Gln on intestinal development (*P* < 0.01). Gln1 and Gln2 groups had higher duodenal VH and V/C, and the Gln2 group increased jejunal VH and V/C, and ileal VH at 21d (*P* < 0.05). Duodenal V/C improved in the Gln1 and Gln2 groups, and duodenal CD reduced in Gln1 group at 42d (*P* < 0.05). Gln1 and Gln2 groups increased cecal α-diversity (ACE, Chao1) at d21 (*P* < 0.05). PCoA revealed distinct cecal microbiota profiles across groups, with Gln-supplemented groups reducing pathogenic *Escherichia-Shigella* and *Mucispirillum*, while enriching beneficial genera (e.g., *Ruminococcaceae_UCG-014, Barnesiella*, and *Butyricimonas*) (*P* < 0.05). Collectively, dietary Gln-supplemented at 4-6 g/kg optimally enhances growth, immunity, and intestinal development in broilers, which is related to modulate cecal microbiota toward a beneficial composition.

## Introduction

The global broiler industry, driven by escalating demands for high-quality animal protein, faces significant challenges in maintaining the broiler health and productivity under intensive farming practices (Cara et al., 2025; Ding et al., 2024). Modern broilers often exhibit compromised immune resilience and intestinal integrity, as well as susceptibility to inflammation and infection by various factors such as feeding practices, environmental stress, and pathogenic invasion (Korver, 2023). In this context, the gastrointestinal tract (GIT), serving as a primary site for nutrient absorption, immune modulation, and microbial balance, plays a crucial role in maintaining animal health and performance (Naithani et al., 2024; Wang et al., 2019a). Gut health is vital for broilers, highlighted by the fact that over half of the antibiotics used therapeutically in the European Union are deployed to control gut-related diseases (Persoons et al., 2012). Disruptions in intestinal morphology, such as villus atrophy, crypt hyperplasia, and mucosal barrier dysfunction, are strongly correlated with diminished nutrient utilization, chronic inflammation, and susceptibility to enteric diseases (Lee and Kim, 2020). Consequently, nutritional interventions targeting intestinal health and immune modulation have emerged as critical strategies to enhance the well-being and production performance of broilers.

Among various nutritional candidates, glutamine (Gln), as a conditionally essential and functional amino acid, has recently received increasing attention due to its dual role as a metabolic substrate and an immune modulator (Li et al., 2023; Liu et al., 2024; Wu et al., 2022). Glutamine serves as a primary energy source for the rapid proliferation of intestinal epithelial cells and immune cells (Wang et al., 2014), particularly under environment stress. Exogenous Gln supplementation could maintain normal cellular physiological functions and growth metabolism. Studies have indicated that supplementing broiler diets with Gln improves their growth performance and reduces stress-related damage in both cold and heat stress conditions (Shakeri et al., 2014; Wu et al., 2025). The mechanisms underlying these benefits are multifaceted. Specifically, dietary Gln has been reported to improve intestinal barrier function and immune homeostasis to promote intestinal health status. Studies demonstrated that dietary Gln supplementation improve intestinal structure by increasing the ratio of villus height to crypt depth and strengthens intestinal barrier function through the upregulation of mucin and tight junction proteins expression (Wu et al., 2018). Concurrently, Gln modulates the immune system by regulating cytokine production (such as IL-10, TNF-α), enhancing macrophage phagocytosis, and reducing oxidative stress (Wu et al., 2021). For instance, dietary Gln supplementation has been shown to improve intestinal immune barrier function in broiler chickens exposed to lipopolysaccharide (LPS) or *Salmonella Enteritidis*, thereby improving growth performance and intestinal mucosal barrier integrity (Wu et al., 2022; Zhang et al., 2022).

Beyond its direct effects on host cells, the potential influence of Gln on the gut microbiota represents another critical avenue through which it may confer health benefits. The gut microbiota plays an essential role in host health by producing short-chain fatty acids (SCFAs), regulating intestinal barrier and immune functions, and competing with intestinal pathogens for colonization (Gao et al., 2022; Rooks and Garrett, 2016). Emerging evidence suggests that Gln can modulate the gut microbiota microbial ecosystem. For example, Gln has been shown to reduce the Firmicutes/Bacteroidetes ratio, activate NF-κB and PI3K-Akt pathways, and inhibit pathogenic bacterial colonization and translocation (Perna et al., 2019). In broilers, a study has been reported that adding combined amino acids (threonine, arginine, and glutamine) to diets could reshape the cecal microbiota profile (Hussein et al., 2023). Furthermore, research in piglets demonstrated that dietary Gly-Gln supplementation enhance intestinal integrity, reduces inflammatory, and improves oxidative status. These benefits are linked to an increase in obligate anaerobes and SCFA-producing bacteria, resulting in an improved gut microbiota and elevated levels of propionic and butyric acids (Xu et al., 2021). Despite these promising findings, the specific causal links and associations between Gln-induced alterations in the gut microbiota and the subsequent improvements in intestinal development, immune function, and overall health in broilers remains limited in current reports.

Given the established link between the intestinal health and gut microbiota balance, there is a compelling need to systematically investigate whether the beneficial impacts of Gln on broiler intestinal morphology and immunity are mediated through specific changes in the cecal microbial community. We hypothesize that dietary Gln supplementation may enhance intestinal morphology and immune function in broilers by selectively modulating cecal microbiota composition. Therefore, this study aimed to investigate the impact of varying levels of Gln supplementation on growth performance, intestinal histomorphology, immune indices, and cecal microbial community in broilers.

## Materials and methods

### Experimental design, diets, and bird management

All experimental procedures were reviewed and approved by the Animal Ethics Committee of Poultry Institute of Shandong Academy of Agricultural Sciences (JQS-2023-G25). The study employed a factorial arrangement of treatments in a completely randomized design. A total of 300 one-day-age healthy male Arbor Acres broilers with similar initial body weight (51.67±1.42 g) were randomly distributed to 5 groups in a completely random design, with 6 replicates each group and 10 broilers in each replicate. Five diets including corn-soybean meal basal diet (Con), four dietary Gln inclusion diets containing 4 g/kg (Gln1), 6 g/kg (Gln2), 8 g/kg (Gln3), and 10 g/kg Gln (Gln4), were allocated randomly to each of groups. Gln, with a purity greater than 99.0 %, was obtained from Jiangxi Guanyin Biotechnology Co., Ltd. (Nanchang, China). The basal diet was formulated to meet or exceeded the nutritional requirements for broilers (NY/T 33-2004). The crude protein, Ca, Lys, and Met contents in diet were determined according to GB/T 6432-2018, GB/T 6436-2018, and GB/T 18246-2019, respectively. Gln content in experimental diets were tested according to previously described method (Dai et al., 2014). In brief, diet samples were conducted to acidic hydrolysis, then high-performance liquid chromatography (HPLC) method was used to determine the Gln content. The ingredients and nutrient levels of basal diet are presented in Table 1.

**Table 1:** Composition and nutrient levels of basal diets (as-fed basis).

Ingredients	Content, %									
	1 to 21 days of age					22 to 42 days of age				
Corn	54.5					60.0				
Soybean meal	33.96					28.26				
Fish meal	3.00					3.60				
Soybean oil	3.50					3.80				
DL-Met	0.200					0.100				
Lys	0.140					0				
Zeolite powder	2.70					2.24				
Premix^1^	2.00					2.00				
Total	100.0					100.0				
Nutrient levels^2^										
ME/(MJ/kg)	12.65					12.99				
CP	21.80					20.02				
Ca	1.00					0.900				
AP	0.450					0.400				
Met	0.520					0.400				
Lys	1.200					1.000				
	Con	Gln1	Gln2	Gln3	Gln4	Con	Gln1	Gln2	Gln3	Gln4
Gln	3.27	3.62	3.85	4.11	4.33	2.91	3.23	3.46	3.75	3.93

1 The premix provided the following per kg of diets: VA 13 000 IU, VB_1_ 3.6 mg, VB_2_ 11.25 mg, VB_6_ 6.0 mg, VB_12_ 0.04 mg, VD_3_ 3 600 IU, VE 36 IU, VK_3_ 4.5 mg, biotin 0.24 mg, folic acid 2.10 mg, d-pantothenic acid 16.50 mg, nicotinic acid 39.2 mg, Cu (as copper sulfate) 16 mg, Fe (as ferrous sulfate) 80 mg, Mn (as manganese sulfate) 120 mg, Zn (as zinc sulfate) 110 mg, I (as potassium iodide) 1.50 mg, Se (as sodium selenite) 0.30 mg..

2 ME and AP were calculated values based on Tables of Feed Composition and Nutritive Values in China (33th Edition, 2022).

The broilers were housed in three-tier pens (150 cm × 90 cm × 60 cm per cell), with ad libitum access to feed and water. The first 3 days, 24 h continuous light was provided, followed by 16 h continuous light and 8 h of darkness. The room temperature was initially kept at 33-35°C during the first 3 days, then gradually lowered to 26°C by 21d. After 21 days, the temperature remained stable at 24-26°C until the end of the experiment.

### Growth performance and sample collection

The feed intake and the body weight of broilers were measured based on a replicate at 21 and 42 days of age following an overnight fast, and the average individual body weight (BW), average daily feed intake (ADFI), average daily gain (ADG), and feed-gain ratio (F/G) were calculated (Lv et al., 2025).

On days 21 and 42, six birds (one per replicate) were randomly selected from each treatment group for sample collections. Blood were sampled from wing veins and centrifuged to detect the concentrations of serum immunoglobulins, after which the birds were euthanized via jugular vein exsanguination. Immune organs including spleen, thymus, and bursa of Fabricius were excised and weighed. The immune organ index (%) = organ weight (g)/body weight (g) × 100 (Yang et al., 2020). The sections (approximately 1 cm) from the middle of duodenum, jejunum, and ileum were sampled and fixed in 4 % paraformaldehyde for intestinal morphological examination. The cecal contents of each broiler were collected in 2 mL tubes, quickly frozen in liquid nitrogen, and kept at −80°C for 16S rDNA sequencing analysis.

### Assessment of serum immune indices

The serum concentrations of immunoglobulin A (IgA), G (IgG), and M (IgM) were measured by an enzyme-linked immunosorbent assay (ELISA). The assays employed commercial kits from Beinglay Biotechnology Co., Ltd. (Wuhan, China), with all procedures strictly adhering to the manufacturer’s protocols.

### Intestinal morphological analysis

The intestinal morphology was analyzed according to our previous study (Lv et al., 2025). The intestinal morphology of duodenum, jejunum, and ileum fixed with 4 % paraformaldehyde was assessed using paraffin slices. The samples processed through dehydrated, paraffin-embedded, sectioned (5 um), and stained with hematoxylin and eosin (H&E). The stained slides were examined using a microscope. Villus height (VH) and crypt depth (CD) of each intestinal segment were measured using Image-Pro Plus 6.0 (Media Cybernetics, Inc., Rockville, MD, USA), and ten villus-crypt pairs per section were analyzed to calculate villus height/crypt depth (V/C) values.

### 16S rRNA gene sequencing and bioinformatic analysis

The bacterial DNA was extracted from cecal contents using the Qiagen DNA isolation kit (Qiagen, Hilden, Germany) following the manufacturers’ protocol. The V3-V4 hypervariable regions of the bacterial 16S rRNA gene was amplified with gene-specific primers F348 (5′-ACTCCTACGGGRSGCAGCAG-3′) and R806 (5′-GGACTACVVGGGTATCTAATC-3′). The purified amplicons were sequenced using paired-end technology on the Illumina HiSeq250 platform (Illumina, San Diego, USA). The 16S rRNA gene microbiome sequencing data was analyzed using BMK Cloud (www.biocloud.net). The raw data were demultiplexed and quality-filtered using QIIME (version 1.9.1). UCLUST in QIIME (version 1.9.1) was used to cluster operational taxonomic units (OTUs) at 97 % similarity, followed by chimera removal with UCHIME (version 7.1). Representative OTU sequences were selected using the GreenGene v13.8 Database. The Wilcoxon rank-sum test was used to analyze the differences in α-diversity indices (Sobs, ACE, Shannon, and Chao1) among the groups. The principal coordinate analysis (PCoA) and PLS-DA analysis with ANOSIM test was performed to establish β-diversity. To determine significant differences among the five groups, a Kruskal-Wallis test was conducted, followed by a Welch’s test, with P < 0.05 showing statistical significance. Linear discriminant analysis and effect size measurements (LEfSe) was further employed to identify the differential genera in each Gln-supplemented group compared to Con group, and *P*-values < 0.05, LDA scores ≥ 3.0 were selected for plotting and further analysis.

### Statistical analysis

An analysis of variance with Tukey's HSD multiple range test was conducted for growth performance, serum immune indices, immune organ index, and intestinal morphology to determine the differences among diet treatments using the GLM procedure of SAS 8.0 (SAS Institute, Inc., Cary, NC, USA). Linear and quadratic effects of the different graded Gln supplementation were also determined using GLM procedure of SAS. The data from a replicate pen was calculated as the experimental unit for the growth performance evaluation. The bird individual from each pen was regarded as the basic unit for other measured parameters. The dietary treatment was independent variable according to the GLM model:

$$Y_{\text{ij}}=\mu +D_{i}+e_{\text{ij}},$$

where Y_ij_ represents the dependent variable, μ represents general mean, D_i_ represents the effect of diet (*i* = Con, Gln1, Gln2, Gln3 and Gln4 group), e_ij_ represents the residual error. Significance was defined as *P* < 0.05.

## Results

### Dietary Gln supplementation improves growth performance of broilers

We initially assessed the effects of graded levels of Gln supplementation on broiler growth performance (Table 2). Compared with the Con group, the Gln-supplemented groups had no significant differences in BW, ADFI, ADG, and F/G of broilers from day 0 to day 21 (P > 0.05). Dietary Gln supplementation had quadratic effect on ADG, F/G, and final BW of broilers during the 22-42 day and 1-42 day (*P* < 0.05). Specifically, the Gln2 group increased ADG compared to the Con group during 22-42d and 1-42 day (*P* < 0.05). The Gln2 groups had higher BW compared to the Con group over 1-42 day (*P* < 0.05).

**Table 2:** Effects of dietary Gln supplementation on growth performance of broilers.

Items	Con	Gln1	Gln2	Gln3	Gln4	SEM	*P*-value	Liner	Quadratic
**1 to 21 days of age**									
ADFI, g	49.5	49.6	49.5	49.7	49.4	0.855	0.990	0.981	0.879
ADG, g	35.5	36.9	37.3	36.6	36.6	0.550	0.273	0.444	0.106
F/G	1.39	1.35	1.33	1.36	1.35	0.033	0.776	0.658	0.396
BW of d21, g	797	826	834	821	820	0.011	0.302	0.390	0.123
**22 to 42 days of age**									
ADFI, g	138	141	143	138	137	2.68	0.536	0.401	0.213
ADG, g	79.2^b^	83.8^ab^	85.5^a^	81.4^ab^	80.0^ab^	1.53	0.039	0.619	0.009
F/G	1.75	1.68	1.67	1.70	1.72	0.023	0.198	0.560	0.038
BW of d42, kg	2.38^b^	2.50^ab^	2.54^a^	2.45^ab^	2.42^ab^	0.034	0.018	0.927	0.005
**1 to 42 days of age**									
ADFI, g	92.9	94.3	95.1	93.0	92.2	1.467	0.632	0.450	0.247
ADG, g	56.8^b^	59.8^ab^	60.8^a^	58.5^ab^	57.8^ab^	0.830	0.018	0.851	0.005
F/G	1.64	1.58	1.56	1.59	1.60	0.018	0.090	0.369	0.026

In the same row, values with different small letter superscripts mean significant difference (*P* < 0.05). The same as following tables.

### Dietary Gln supplementation elevates serum immune indices of broilers

The concentrations of serum immune indices of 21- and 42-day-old broilers were assessed (Table 3). Compared with the Con group, all Gln-supplemented groups increased the serum IgA levels in 21- and 42-day-old broilers (*P* < 0.05). Dietary Gln supplementation had quadratic effect on IgM in 21-day-old broilers, and on IgG in 21- and 42-day-old broilers (*P* < 0.01). The Gln1, Gln2 and Gln3 groups increased serum IgG levels in 21-day-old broilers (*P* < 0.05), and serum IgM levels in 42-day-old broilers (*P* < 0.05). The Gln1 and Gln2 groups increased serum IgM levels in 21-day-old broilers (*P* < 0.05), and serum IgG levels in 42-day-old broilers (*P* < 0.05).

**Table 3:** Effects of dietary Gln supplementation on serum immune indicators of broilers.

Items	Con	Gln1	Gln2	Gln3	Gln4	SEM	*P*-value	Liner	Quadratic
**21d of age**									
IgG, μg/mL	14.8^d^	18.2^b^	20.3^a^	17.0^bc^	15.3^cd^	0.385	<0.001	0.088	<0.001
IgA, μg/ml	41.3^c^	57.6^a^	56.9^a^	53.9^a^	47.2^b^	1.12	<0.001	0.033	<0.001
IgM, ng/ml	282^d^	431^a^	352^b^	297^cd^	322^c^	6.12	<0.001	0.214	<0.001
**42d of age**									
IgG, μg/mL	17.7^b^	23.4^a^	22.4^a^	18.9^b^	18.6^b^	0.369	<0.001	0.164	<0.001
IgA, μg/ml	48.6^d^	72.8^b^	79.9^a^	64.4^c^	63.8^c^	0.981	<0.001	0.001	<0.001
IgM, ng/ml	265^c^	301^b^	433^a^	313^b^	291^bc^	5.75	<0.001	0.004	<0.001

### Dietary Gln supplementation promotes the development of immune organs in broilers

The immune organ index, including thymus, spleen and bursa of Fabricius were also measured in 21- and 42-day-old broilers (Table 4). Dietary Gln supplementation had quadratic effect on thymus index in 21-day-old broilers, and bursa of Fabricius index in 21- and 42-day-old broilers (*P* < 0.01). The bursa of Fabricius index was higher in the Gln1 and Gln2 groups compared to the Con group in both 21- and 42-day-old broilers (*P* < 0.05). The Gln2 group showed improvement in thymus index in 42-day-old broilers compared to the Con group (*P* < 0.05).

**Table 4:** Effects of dietary Gln supplementation on immune organ index of broilers.

Items	Con	Gln1		Gln2		Gln3	Gln4	SEM	*P*-value	Liner	Quadratic
**21d of age**											
Thymus index, g/kg	3.75	4.43		4.27		3.77	3.64	0.461	0.674	0.629	0.230
Spleen index, g/kg	0.860	1.08		1.06		0.927	0.949	0.085	0.364	0.874	0.118
Bursa of Fabricius index, g/kg	2.40^b^	3.06^a^		3.09^a^		2.64^ab^	2.69^ab^	0.147	0.011	0.767	0.005
**42d of age**											
Thymus index, g/kg	3.46^b^	4.08^ab^		4.98^a^		3.90^ab^	3.53^b^	0.237	<0.001	0.225	0.002
Spleen index, g/kg	1.15	1.33		1.40		1.25	1.27	0.082	0.274	0.735	0.127
Bursa of Fabricius index, g/kg	1.56^b^	2.08^a^		2.09^a^		1.81^ab^	1.88^ab^	0.116	0.019	0.333	0.011

### Dietary Gln supplementation enhances the intestinal development in broilers

We next explored the impact of the Gln supplementation on intestinal morphology in broilers (Table 5). At 21 day of age, dietary Gln supplementation had quadratic effect on the duodenal VH (*P* < 0.01), with higher duodenal V/C in the Gln1 and Gln2 groups compared to the Con group (*P* < 0.05). All Gln-supplemented groups increased duodenal V/C (*P* < 0.05). Dietary Gln supplementation had quadratic effect on jejunal and ileal VH (*P* < 0.01), which higher in the Gln2 group (*P* < 0.05). Additionally, the Gln2 group also increased jejunal V/C (*P* < 0.05). Supplementing Gln in the diet quadratically improved in ileal V/C (*P* < 0.01). At 42 days of age, dietary supplementation with Gln had no significant effect on duodenal VH, jejunal and ileal VH, CD, V/C compared to the Con group (*P* > 0.05). There was quadratic effect of Gln on duodenal CD and V/C of broilers (*P* < 0.01), with duodenal CD decreased in the Gln1 group, while duodenal V/C increased in the Gln1 and Gln2 groups (*P* < 0.05).

**Table 5:** Effects of dietary Gln supplementation on intestinal morphology of broilers.

Items	Con	Gln1	Gln2	Gln3	Gln4	SEM	*P*-value	Liner	Quadratic
**21d of age**									
Duodenum									
VH/μm	1184^b^	1366^a^	1401^a^	1332^ab^	1297^ab^	37.4	0.004	0.118	0.001
CD/μm	144	134	129	134	133	4.80	0.286	0.188	0.118
V/C	8.30^b^	10.2^a^	10.9^a^	9.96^a^	9.80^a^	0.316	<0.001	0.012	<0.001
Jejunum									
VH/μm	784^b^	871^ab^	956^a^	827^ab^	865^ab^	33.9	0.028	0.286	0.025
CD/μm	122	125	122	123	119	4.96	0.944	0.654	0.626
V/C	6.41^b^	6.99^ab^	7.84^a^	6.78^ab^	7.30^ab^	0.287	0.026	0.101	0.075
Ileum									
VH/μm	588^b^	709^ab^	783^a^	693^ab^	672^ab^	40.2	0.029	0.232	0.006
CD/μm	111	117	122	118	119	4.57	0.528	0.241	0.272
V/C	5.28	6.07	6.43	5.88	5.67	0.269	0.055	0.481	0.007
**42d of age**									
Duodenum									
VH/μm	1436	1594	1704	1567	1595	79.3	0.232	0.254	0.099
CD/μm	223^a^	175^b^	183^ab^	183^ab^	210^ab^	10.8	0.018	0.614	0.002
V/C	6.57^b^	9.23^a^	9.42^a^	8.56^ab^	7.62^ab^	0.515	0.003	0.384	<0.001
Jejunum									
VH/μm	1165	1214	1239	1199	1124	56.4	0.647	0.591	0.152
CD/μm	203	175	177	183	173	10.2	0.249	0.110	0.316
V/C	5.86	6.95	7.10	6.66	6.53	0.422	0.298	0.443	0.067
Ileum									
VH/μm	768	831	910	803	821	37.7	0.138	0.517	0.062
CD/μm	150	141	158	145	143	9.20	0.719	0.749	0.669
V/C	5.15	5.99	5.8	5.63	5.73	0.233	0.162	0.298	0.108

### Dietary Gln supplementation modulates cecal microbiota of broilers

**Fig. 1:**
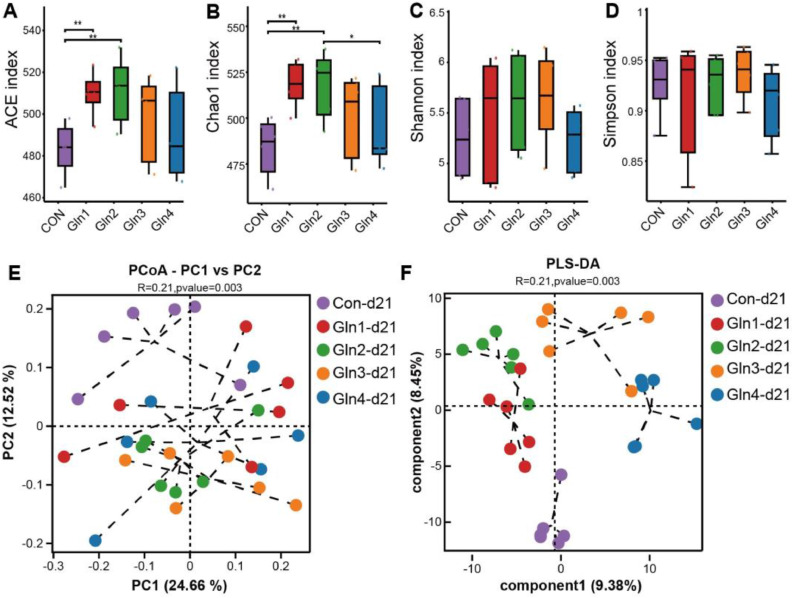
Effects of dietary Gln supplementation on diversity of cecal microbiota at day 21. (A) Ace index, (B) Chao1 index, (C) Shannon index, (D) Simpson index, (E) Principal coordinate analysis (PCoA) plot and (F) PLS-DA of cecal microbiota composition. * indicates *P* < 0.05, ** indicates *P* < 0.01.

**Fig. 2:**
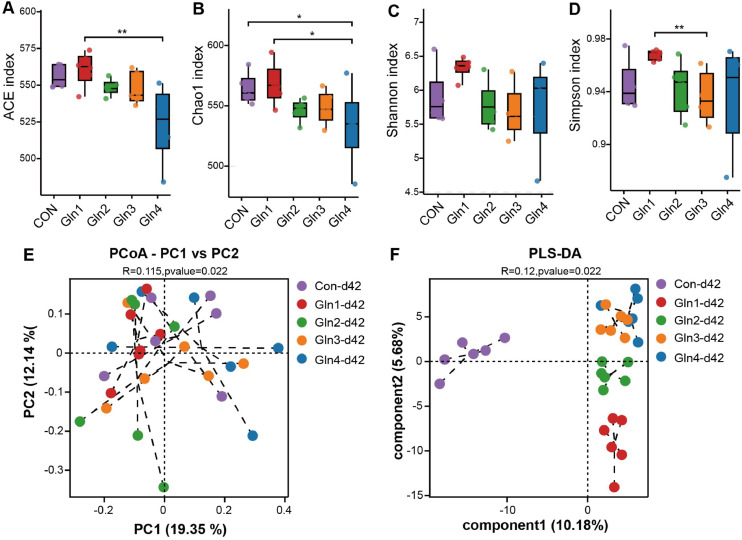
Effects of dietary Gln supplementation on diversity of cecal microbiota at day 42. (A) Ace index, (B) Chao1 index, (C) Shannon index, (D) Simpson index, (E) Principal coordinate analysis (PCoA) plot and (F) PLS-DA of cecal microbiota composition. * indicates *P* < 0.05, ** indicates *P* < 0.01.

16S rDNA amplicon sequencing was conducted to investigate the alterations in the diversity and composition of cecal microbiota. At 21 day of age, Gln1 and Gln2 groups improved ACE and Chao1 index compared to Con group (*P* < 0.01, Fig. 1A, B). Whereas, dietary Gln supplementation did not significantly affect the Shannon and Simpson indices (*P* > 0.05, Fig. 1C, D). In addition, Gln2 group had higher Chao1 index than Gln4 group (*P* < 0.05, Fig. 1B). The PCoA analysis revealed a distinct separation in microbial composition between the Con group and the four Gln-supplemented groups (*P* = 0.003, Fig. 1E). The PLS-DA revealed significant clustering differences among the groups (*P* = 0.003, Fig. 1F). At 42 day of age, dietary Gln supplementation did not significantly affect ACE, Shannon, and Simpson indices (*P* > 0.05, Fig. 2A, C, and D), whereas, the Gln4 group showed a decrease in the Chao1 index compared to the Con group (*P* < 0.05, Fig. 2B). Additionally, Gln1 group has higher α-diversity (ACE, Chao1, and Simpson index) than Gln3 or Gln4 groups (*P* < 0.05, Fig. 2A, B, and D). The PCoA and PLS-DA indicated that the cecal microbial composition of broilers had different cluster among groups (*P* = 0.022, Fig. 2E, F).

**Fig. 3:**
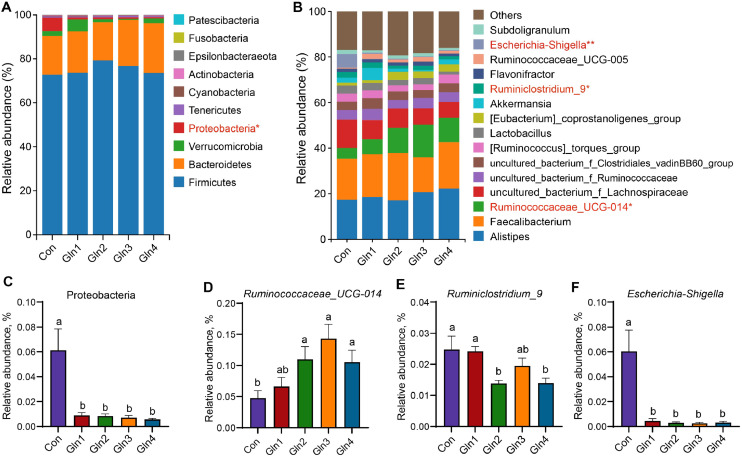
Effects of dietary Gln supplementation on cecal microbiota compsosition at day 21. Relative abundances of cecal microbiota at phylum level (A) and genus level (B), and differential bacteria were detected by the Wilcoxon rank sum test at phylum (C) and genus (D-F) level. * indicates *P* < 0.05, ** indicates *P* < 0.01.

**Fig. 4:**
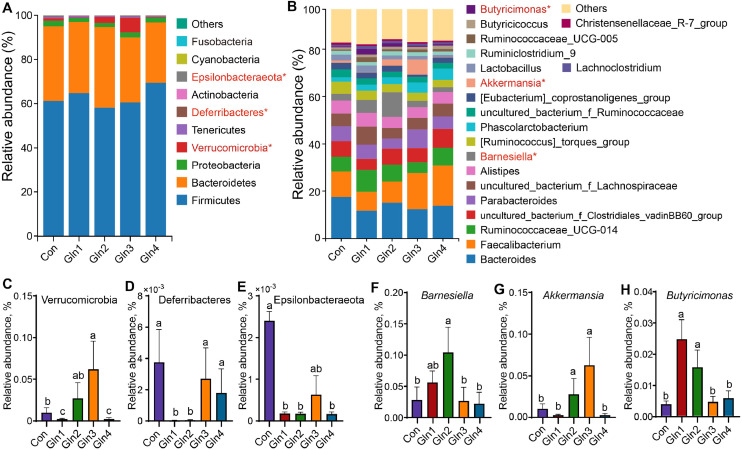
Effects of dietary Gln supplementation on cecal microbiota compsosition at day 42. (A) Relative abundances of cecal microbiota at phylum level (A) and genus level (B). Differential bacteria at phylum (C-E) and genus (F-H) level detected by the Wilcoxon rank sum test. * indicates *P* < 0.05.

Taxonomic analysis indicated that at 21 day of age, there were 9 bacterial phyla, and Firmicutes (75.23 %) and Bacteroidetes (19.45 %) predominated the cecal microbiota (Fig. 3A) at the phylum level. The Gln-supplemented groups significantly decreased the abundance of Proteobacteria (*P* < 0.05, Fig. 3C). At the genus level, the average abundance of the top 15 genera of each group are presented in Fig. 3B, which accounted for 83.97 %. Dietary Gln-supplemented groups, except for Gln1 group, showed an increased abundance of *Ruminococcaceae_UCG-014* (*P* < 0.05, Fig. 3D). Gln2 and Gln4 groups showed a decreased abundance of *Ruminiclostridium_9* (*P* < 0.05, Fig. 3E). Dietary Gln supplementation decreased the abundance of *Escherichia-Shigella* (*P* < 0.05, Fig. 3F). At 42 day of age, there were 11 bacterial phyla, and Firmicutes (62.90 %) and Bacteroidetes (31.84 %) predominated the cecal microbiota at the phylum level (Fig. 4A). Three differential phyla including Verrucomicrobia, Deferribacteres, and Epsilonbacteraeota were detected across groups (*P* < 0.05, Fig. 4C, D, and E). The Gln1 and Gln2 groups significantly decreased the Deferribacteres and Epsilonbacteraeota abundance (*P* < 0.05, Fig. 4D, E). Gln1 and Gln4 groups decreased the Verrucomicrobia abundance (*P* < 0.05, Fig. 4C). At the genus level, the top 20 genera in abundance occupied 83.15 % of the total (Fig. 4B). The Gln2 group increased the abundance of *Barnesiella, Akkermansia* and *Butyricimonas* (*P* < 0.05, Fig. 4F, G, and H). The Gln1 group increased the *Butyricimonas* abundance (*P* < 0.05, Fig. 4H), and Gln3 group increased the *Akkermansia* abundance (*P* < 0.05, Fig. 4G).

**Fig. 5:**
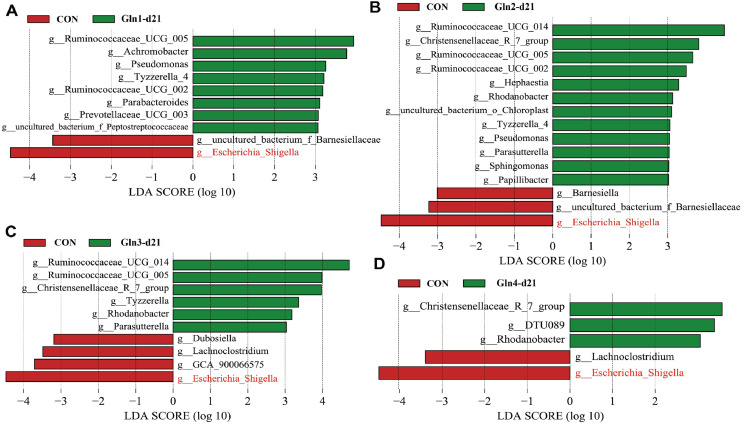
Linear discriminant analysis effect size (LEfSe). Differential bacteria at the genus level between the Con and Gln1 (A), Con and Gln2 (B), Con and Gln3 (C), and Con and Gln4 (D) at d21.

**Fig. 6:**
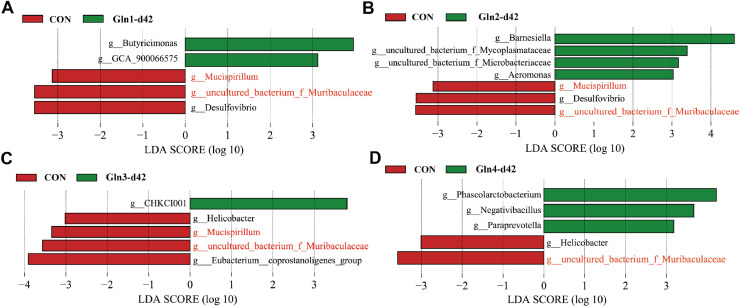
Linear discriminant analysis effect size (LEfSe). Differential bacteria at the genus level between the Con and Gln1 (A), Con and Gln2 (B), Con and Gln3 (C), and Con and Gln4 (D) at d42.

LEfSe analysis was also performed to identify specific bacteria enriched in Gln-supplemented groups and Con group. We found that Gln2 group had the most differential genera compared to Con group at d21 (Fig. 5B). Among those genera, *Ruminococcaceae_UCG-005, Tyzzerella*, and *Parasutterella* were also enriched in Gln1 group (*P* < 0.05, Fig. 5A), *Ruminococcaceae_UCG-014, Ruminococcaceae_UCG-005, Rhodanobacter, Christensenellaceae_R-7_group, Tyzzerella*, and *Parasutterella* were also enriched in Gln3 group (*P* < 0.05, Fig. 5C), and the *Christensenellaceae_R-7_group* and *Rhodanobacter* were also enriched in Gln4 group (*P* < 0.05, Fig. 5D). Notably, Gln supplementation significantly decreased the abundance of *Escherichia-Shigella* (*P* < 0.05, Fig. 5A, B, C, and D). At 42 day of age, the characteristic bacteria were differently enriched in Gln-supplemented groups. The *Butyricimonas* and *GCA_900066575* were enriched in Gln1 group (*P* < 0.05, Fig. 6A), the *uncultured_bacterium_f_Mycoplasmataceae, Barnesiella, Aeromonas*, and *uncultured_bacterium_f_Microbacteriaceae* were enriched in Gln2 group (*P* < 0.05, Fig. 6B), the *CHKCI001* was enriched in Gln3 group (*P* < 0.05, Fig. 6C), and the *Phascolarctobacterium, Negativibacillus*, and *Paraprevotella* were enriched in Gln4 group (*P* < 0.05, Fig. 6D). Gln supplementation consistently decreased the abundance of *uncultured_bacterium_f_Muribaculaceae* (*P* < 0.05, Fig. 6A-D), and *Mucispirillum* (*P* < 0.05, Fig. 6A-C).

### The growth performance, immune indicators, and intestinal morphology correlate with cecal microbiota

**Fig. 7:**
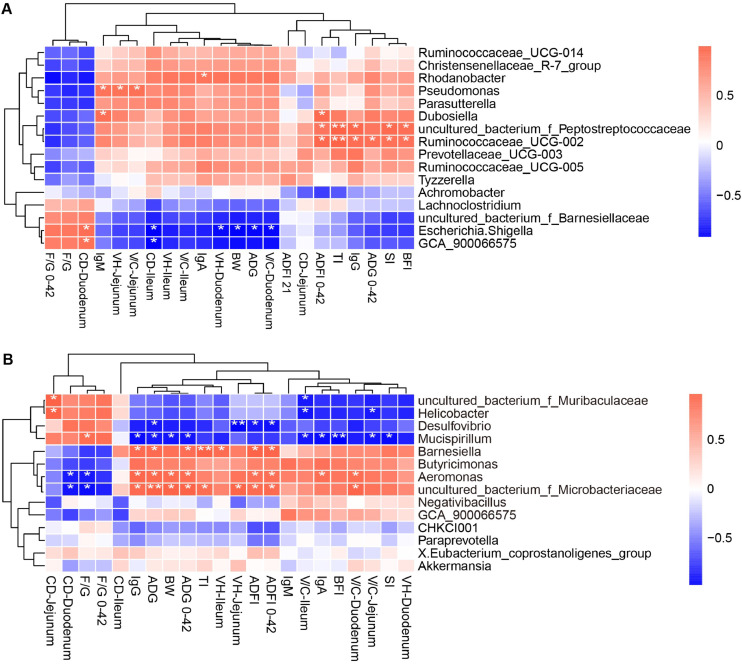
Correlations among cecal microbes, immune indicators, and intestinal morphology. (A) Clustered heatmaps at d21. (B) Clustered heatmaps at d42. * indicates *P* < 0.05, ** indicates *P* < 0.01.

Correlation analyses were performed to explore the relationships between the growth performance, immune indicators, intestinal morphology, and cecal microbes. At 21 day of age, we found that Gln-enriched genura, *Ruminococcaceae_UCG-002, uncultured_bacterium_f_Peptostreptococcaceae* had a positive correlation with ADFI and ADG from 0 to 42, IgG, and immune organ indices (*P* < 0.05, Fig. 7A), *Pseudomonas* had a positive correlation with IgM, jejunal VH and V/C (*P* < 0.05, Fig. 7A), and *Rhodanobacter* had a positive correlation with IgA (*P* < 0.05, Fig. 7A). Whereas, Con-enriched *Escherichia.Shigella* were negatively correlated with BW, ADG, duodenal VH and V/C (*P* < 0.05, Fig. 7A). At 42 day of age, Gln-enriched genura, such as *uncultured_bacterium_f_Microbacteriaceae, Barnesiella*, and *Aeromonas* had a positive correlation with ADFI, ADG from 21 to 42d and 0 to 42d, and IgG levels (*P* < 0.05, Fig. 7B), of which, *Barnesiella* also had a positive correlation with ileal VH and thymus index (*P* < 0.05, Fig. 7B), and *uncultured_bacterium_f_Microbacteriaceae* also had a positive correlation with BW, thymus index, jejunal VH, and duodenal V/C (*P* < 0.05, Fig. 7B). Con-enriched *Mucispirillum* showed a negative correlation with BW, ADG, immunoglobulins IgA and IgG, organ index, and jejunal and ileal V/C (*P* < 0.05, Fig. 7B), *Desulfovibrio* was negatively associated with ADG, ADFI, jejunal VH (*P* < 0.05, Fig. 7B), and *Helicobacter* was negatively related to jejunal and ileal V/C (*P* < 0.05, Fig. 7B).

## Discussion

Glutamine (Gln) is a crucial energy source that enhances the intestinal cell development, thereby facilitating nutrient absorption and growth. Previously studies have indicated that dietary glutamine supplementation increased the feed consumption and BWG, and decreased F/G in broilers (Bai et al., 2023; Hu et al., 2016; Xue et al., 2018). However, high levels of Gln addition (4 %) depressed growth performance of broilers (Bartell and Batal, 2007). The above results indicated that there is a dose-dependent effect of dietary Gln supplementation on animal growth performance. In the present study, we found that dietary Gln supplementation with 4-6 g/kg optimally enhanced growth performance of broilers. Our findings of an optimal supplementation range for broiler growth performance clearly demonstrate a dose-dependent effect of dietary Gln supplementation. In line with our result, a study on piglets also demonstrated that a diet containing 0.4 % Gln was the most beneficial for growth performance among diets with 0.2 %−1.0 % Gln (Duttlinger et al., 2020). This phenomenon may be attributed to the fact that high concentrations of Gln could not be metabolized by animals (Li et al., 2024), and might cause antagonism with other amino acids such as arginine and proline, thereby inhibiting growth performance (Holecek, 2013). Under stress conditions, the effective dosage of glutamine required by animals was increased. Studies have demonstrated that dietary glutamine supplementation reached 1 %−1.5 % could improve growth performance in animals under heat stress, cold stress, or pathogen challenge conditions (Bai et al., 2023; Liu et al., 2024; Wu et al., 2025).

The intestine functions as both the primary site of nutrient digestion and absorption and a critical immune organ in broilers (Yan et al., 2021). It has reported that Gln improved broiler growth performance by promoting a healthy intestine (Kishawy et al., 2024; Wu et al., 2021). Consistently, our findings indicated that Gln treatment enhanced broiler growth performance, which is closely linked to improved gut function, as demonstrated by enhanced intestinal morphology and immune function. The serum immunoglobulins (eg. IgA, IgM, IgG) are vital to the immune system, with their blood concentrations serving as key indicators of immune function (Lanzarini et al., 2018). Studies reported that Gln could provide the energy source and metabolic precursors needed for immune cell proliferation, and strengthen immune function (Gomes et al., 2022; Johnson and Lay, 2017). The observed elevation in serum IgA, IgM, and IgG levels in broilers supplemented with 4-6 g/kg Gln in our study directly reflects an enhancement of systemic humoral immunity. In agreement, study reported that supplementation with Gln increased serum IgA, IgG, and IgM concentrations of broilers (Wu et al., 2022). Immune organ index plays a critical role in evaluating the developmental status of immune organs, reflecting the organism's capacity to respond to external stress. The thymus, spleen, and bursa of Fabricius serve as pivotal sites for immune cell differentiation and antibody production, constituting the primary immune organs in poultry. Gln provides abundant bioactive precursors, including purines and pyrimidines, to support immune organ development, thereby increasing both cellular and humoral immune functions (Allahdo et al., 2018; Wang et al., 2015). Previous study reported that dietary Gln supplementation elevated the relative weights of the spleen and bursa in chickens (Wu et al., 2021). This study demonstrated that supplementing chicken diets with 4-6 g/kg Gln increased the bursa index and thymus index in broilers, indicating that Gln enhances immune organ development and strengthens resistance against pathogenic infections and stressful challenges.

The small intestine morphology of villi reflects the physiological and functional state of intestine, and plays an important role in maintaining the nutrient absorption and intestine immune (Dong et al., 2016). Longer villi create more favorable conditions for absorbing nutrients (Wang et al., 2022). The improvements in jejunal and ileal VH and V/C observed in our study, particularly with 6 g/kg Gln supplementation, provide structural evidence for enhanced nutrient absorption capacity. Studies have reported that the addition of Gln provides energy for the growth of intestinal epithelial cells, thereby promoting the growth and development of the intestinal tract, with increasing the villus height and V/C of the small intestine in broilers (Wu et al., 2018; Zhang et al., 2022). The results above demonstrated that Gln positively influences epithelial cell growth and proliferation, which is beneficial for sustaining and improving the intestinal microstructure of broilers.

Beyond the intestinal mucosa, the cecal microbiota represents another critical component of gut health that was positively modulated by Gln (Ma and Ma, 2019; Perna et al., 2019). In this study, we found that 4-6 g/kg Gln supplementation increased the cecal microbial α-diversity (ACE and Chao1 indexes) at d21. This early enhancement of microbial diversity suggests that Gln contributes to establishing a more complex and stable microbial ecosystem during a crucial developmental period. This may be attributed to the immature development of both the intestinal tract and microbial ecosystem during the early growth stage (Yang et al., 2022), where Gln promoted beneficial bacterial populations while inhibiting harmful bacteria. It has been reported that the early growth period of broilers is a critical time for nutritional intervention to modify microbial composition (Gong et al., 2019; Zhang et al., 2025). In agreement, studies reported that piglets fed a Gln-supplemented diet increased beneficial bacteria, including *Gemmiger, Lactobacillus*, while decreased the decreased harmful bacteria, like *Clostridium_sensu_stricto-1* and *Streptococcus* (Li et al., 2024; Liu et al., 2024). Furthermore, we found the dietary Gln supplementation altered the composition and structure of cecal microbiota in broilers. Aligned with our findings, studies on piglets and rats have demonstrated that Gln could effectively modulate the intestinal microbiota composition (He et al., 2022; Liu et al., 2024). This modulation was characterized by a reduction in potentially pathogenic taxa and an enrichment of beneficial genera. Proteobacteria are spoilage bacteria, which can disrupt gastrointestinal function and leading to various diseases such as metabolic disorders and IBD (Yu et al., 2022; Zeng et al., 2017). *Escherichia-Shigella* is known as an opportunistic pathogen, which can induce necrotizing enterocolitis and intestinal peristalsis inhibition (Wang et al., 2019b). In this study, the reduction in the levels of Proteobacteria and the opportunistic pathogen *Escherichia-Shigella*, alongside the increase in beneficial genera like *Ruminococcaceae_UCG-014*, points towards a shift in the microbial balance away from potential pathogens and towards taxa associated with gut health. LEfSe analysis showed 6 g/kg Gln supplementation had the most differential genera, in which *Ruminococcaceae_UCG-005, Tyzzerella, and Parasutterella* were also enriched in Gln1 group (4 g/kg). *Ruminococcaceae* is a key bacterial group responsible for fiber degradation SCFAs production (Chen et al., 2021), which play a role in maintaining intestinal health. *Parasutterella*, linked to the fatty acid biosynthesis pathway, might be important for body weight gain (Henneke et al., 2022), aligning with our findings that 4-6 g/kg Gln supplementation enhanced broiler growth performance. Studies found that the abundance of Deferribacteres was increased in inflammation and microbial dysbiosis mice (Terzo et al., 2020), and the Epsilonbacteraeota abundance was increased in DSS mice (Wang et al., 2020), which indicating the Deferribacteres and Epsilonbacteraeota abundance were positively related to intestinal inflammation. At age of 42 days, the modulatory effects of Gln persisted, characterized by a decrease in phyla like Deferribacteres and Epsilonbacteraeota, which have been associated with intestinal inflammation. Conversely, we observed an increase in beneficial genera such as *Barnesiella, Akkermansia, and Butyricimonas*. The enrichment of these taxa, known for their anti-inflammatory properties and roles in maintaining gut barrier integrity, suggests that Gln fosters a microbiota profile conducive to long-term intestinal homeostasis. LEfSe analysis indicated that broilers fed diet with 4-6 g/kg Gln enriched *Butyricimonas, Barnesiella*, while broilers fed basal diet enriched *Mucispirillum*, and *uncultured_bacterium_f_Muribaculaceae*. Study demonstrated that the genus *Barnesiella* is the most abundant genus in healthy gut microbiome, and have anti-inflammatory protection in mice (Weiss et al., 2014). *Butyricimonas* is known as butyrate-producing bacteria with anti-inflammatory effects (den Besten et al., 2013). *Akkermansia*, known for degrading mucin, produces metabolites that provide energize butyrate-producing intestinal bacteria in the gut, which contribute to numerous beneficial functions such as reducing inflammation, improving gut barrier integrity, and modulating the immune system (Cani et al., 2022). *Mucispirillum* can be considered as opportunistic pathogens, and a study has proved that *Mucispirillum* was associated with a greater risk of intestinal inflammation (Huang et al., 2021). A previous study found that the *uncultured_bacterium_f_Muribaculaceae* had a negative correlation with intestinal mRNA expression of claudin-1 and occludin, indicating it may reduce gut barrier function (Azad et al., 2022). These findings indicated that Gln altered the cecal microbial community in broilers by enhancing beneficial bacteria and diminishing harmful ones.

## Conclusions

In conclusion, dietary supplementation with 4-6 g/kg Gln enhanced broiler growth performance, systemic immunity, and intestinal health. The beneficial effects were particularly linked to an early increase in microbial diversity and a regulate in microbial composition by enriching beneficial genera (e.g., *Ruminococcaceae_UCG-014, Barnesiella, Butyricimonas*) and suppressing pathogens (*Escherichia-Shigella, Mucispirillum*). These findings support that Gln has potential as an effective antibiotic alternative for sustainable poultry production. The precise molecular mechanisms connecting Gln-induced microbial changes to host immune and metabolic functions remain to be fully elucidated and need to be further study.

## CRediT authorship contribution statement

**Xuelan Liu:** Writing – original draft, Software, Methodology, Investigation, Funding acquisition, Formal analysis, Conceptualization. **Shuhang Yin:** Methodology, Investigation. **Chunyan Fu:** Validation, Methodology, Investigation, Funding acquisition, Formal analysis. **Xia Li:** Methodology, Investigation. **Peipei Yan:** Methodology, Investigation. **Heng Zhang:** Validation, Methodology, Data curation. **Yan Shang:** Methodology, Investigation. **Tianhong Shi:** Validation, Methodology, Conceptualization. **Qingtao Gao:** Writing – review & editing, Validation, Methodology, Investigation, Funding acquisition, Data curation, Conceptualization.

## Disclosures

No conflict of interest exists in the submission, and all authors have approved the manuscript.
